# The provision of nutritional advice and care for cancer patients: a UK national survey of healthcare professionals

**DOI:** 10.1007/s00520-020-05736-y

**Published:** 2020-09-12

**Authors:** Jane L. Murphy, Fehmidah Munir, Fiona Davey, Laura Miller, Ramsey Cutress, Rhys White, Megan Lloyd, Justin Roe, Carol Granger, Sorrel Burden, Lesley Turner

**Affiliations:** 1 Faculty of Health & Social Sciences, Bournemouth University, 10 St Paul’s Lane, Bournemouth BH8 8AJ, UK; 2 School of Sport, Exercise & Health Sciences, Loughborough University, Loughborough, UK; 3 NIHR Cancer and Nutrition Collaboration, University Hospital Southampton NHS Foundation Trust, Southampton, UK; 4 Nutrition and Dietetics Department, Nottingham University Hospitals NHS Trust, Nottingham, UK; 5 NIHR Nottingham BRC, Nottingham, UK; 6 University Hospital Southampton NHS Foundation Trust, Southampton, UK; 7 Nutrition and Dietetics, Guys and St Thomas’ NHS Foundation Trust, London, UK; 8 The Royal Marsden NHS Foundation Trust, London, UK; 9 Imperial College Healthcare NHS Trust, London, UK; 10 Imperial College, London, UK; 11 Penny Brohn Cancer Care, Bristol, UK; 12 School of Health Sciences, University of Manchester, Manchester, UK

**Keywords:** Cancer, Nutrition, Healthcare professional, Nutritional advice, Guidelines, Survey

## Abstract

**Purpose:**

People living with and beyond cancer often experience nutrition-related issues and should receive appropriate advice on nutrition that is consistent and evidence based. The aim of this study was to investigate current practice for the provision of nutritional care by healthcare professionals (HCPs) from a UK national survey produced by the National Institute for Health Research (NIHR) Cancer and Nutrition Collaboration.

**Methods:**

An online survey sent to professional groups and networks included questions on discussing nutrition, providing information, awareness of guidelines, confidence in providing nutritional advice, training and strategies for improving nutritional management.

**Results:**

There were 610 HCPs who responded including nurses (31%), dietitians (25%), doctors (31%) and speech and language therapists (9%). The majority of HCPs discusses nutrition (94%) and provides information on nutrition (77%). However, only 39% of HCPs reported being aware of nutritional guidelines, and just 20% were completely confident in providing nutritional advice. Awareness of guidelines varied between the different professional groups with most but not all dietitians reporting the greatest awareness of guidelines and GPs the least (*p =* 0.001). Those HCPs with a greater awareness of guidelines had received training (*p* = 0.001) and were more likely to report complete confidence in providing nutritional advice (*p =* 0.001).

**Conclusion:**

Whilst HCPs discuss nutrition with cancer patients and may provide information, many lack an awareness of guidelines and confidence in providing nutritional advice. To ensure consistency of practice and improvements in patient care, there is scope for enhancing the provision of appropriate nutrition education and training.

## Background

Nutrition is an important determinant of well-being for people living with and beyond cancer, affecting response to therapy and preventing secondary recurrence of cancer and development of other non-communicable diseases [1, 2]. With increased numbers of people being diagnosed and surviving cancer in the UK [3], access to appropriate dietary advice and care is part of the NHS England National Cancer Strategy supported by the NHS in England [4]. International guidelines recommend that cancer patients receive tailored nutrition advice from trained healthcare professionals (HCPs) appropriate for the cancer site, their nutritional status and treatment toxicity [5]. The importance of the ‘teachable moment’ has been described following a diagnosis as an opportunity for individuals to change undesirable diet-related behaviours and adopt risk reducing strategies and manage symptoms [6]. As people living beyond a cancer diagnosis are reported to change eating habits [7], there may be multiple teachable moments throughout a patient’s care journey. This is at a time when individuals are susceptible to receive information from less trusted sources such as the Internet that can increase confusion and anxiety. Therefore, it is crucial that healthcare professionals are able to provide appropriate and consistent advice on nutrition-related issues [8].

However, not all people living with and beyond cancer receive professional advice about nutrition and diet including those who are at risk of malnutrition [5] or people who are diagnosed with cancer and complete treatment [9]. Whilst nutritional advice is valued by people following a cancer diagnosis, they have reported that advice received by healthcare professionals has been inconsistent [10–12].

Oncologists may not reliably identify those at nutritional risk due to lack of knowledge and training, awareness of guidelines, time constraints and lack of standardised protocols for care [13, 14]. The lack of awareness and consideration of nutritional issues was reported in a recent survey of Italian oncologists [15]. A UK survey of specialist nurses, clinicians, surgeons and allied health professionals also showed that only half were aware of diet as part of lifestyle guidelines for people living with and beyond a cancer diagnosis and after completing treatment [16].

Given these findings, there is an urgent need to engage a wide range of HCPs who provide nutritional care and advice for people living with and beyond cancer [17]. In response, the National Institute for Health Research (NIHR) Cancer and Nutrition Collaboration (http://cancerandnutrition.nihr.ac.uk) developed a national UK survey of healthcare professionals to investigate the breadth and diversity of practice. For the purpose of this survey, people diagnosed with cancer were referred to as cancer patients. This paper presents the survey’s findings that aimed to examine the provision of nutrition information by HCPs, their confidence in providing nutritional advice and their awareness of guidelines. It also investigates the training needs of HCPs to inform a future strategic framework for nutrition education and training on nutrition and cancer. Other aspects considered in the survey with respect to assessment of nutritional status and tools used in practice will be presented in a separate paper.

## Methods

### Survey development and measures

A multidisciplinary professional working group was established from known members of the NIHR Cancer and Nutrition collaboration toolkit and professionals work streams to develop the survey tool. The survey component identification included HCP delivery of nutrition information and forms of information sharing, education and confidence in nutrition knowledge and perceived training requirements. Initial survey queries were compiled by a subgroup of toolkit members and circulated to the working group for review. Members assessed questions for clinical and scientific relevance, question construct/interpretation and response variable accuracy. This was done over two teleconferences, and additional comments were taken via email after both meetings. The survey was then piloted with a group of five oncology dietitians and two surgeons from a local hospital. Several oncologists and nurses were also contacted, but a response was not received. Piloting allowed a realistic estimate of time-to-complete, response rate and flagged a small number of ambiguities, which were corrected prior to implementation.

The final 21-item online questionnaire included queries on nutrition assessment and monitoring as well as education/training needs. Here, we report on only the education, training and information provision of HCP (Table 1). The completed survey used mixed response variables including Likert scales, single and multiple responses along with open-ended queries. The final questions were exported to iSurvey, a survey tool of Southampton University.

**Table 1:** Reported survey queries relating to nutrition education, information provision and training needs of healthcare professionals

Topic	Survey question	Responses	Sub-question	Responses
Provision of nutrition advice	Do you provide information on nutrition to cancer patients?	Yes	What do you provide?	Locally produced leaflets/verbal advice/nationally produced leaflets/website/others (free text)
		No		
	How confident do you feel in providing nutritional advice to cancer patients?	Not at all confident/not very confident/neutral/somewhat confident/completely confident		
Awareness of guidelines	Are you aware of any guidelines for nutritional advice for cancer patients?	Yes	What do you use and where does it come from?	Free text
		No		
Training in nutrition	Have you ever received training on nutritional care for cancer patients?	Yes	What type of training and how long ago?	Free text
		No		
	Do you feel that you need further training in relation to nutritional care of cancer patients?	Yes	What training would specifically benefit your clinical practice?	Assessment of nutrition status/artificial feeding/supplements/dietary advice for specific cancer site or stage/physical activity assessment/evidence for alternative dietary approaches/other
		No		
	Which methods of training delivery do you prefer?’	Face-to-face/conference or study day/e-learning/webinars/other		
GP specific questions	Do you feel GPs have a role to play in supporting the nutritional needs of patients living with and beyond cancer?	Free text		

### Participants

The online survey was disseminated to HCPs, with a follow-up reminder via professional groups, networks and personal contacts, including the British Dietetic Association, Royal College of Nursing, UK Oncology Nursing Society, British Association of Surgical Oncology/Association for Cancer Surgery, Royal College of General Practitioners, British Association of Head and Neck Oncologists, British Psychosocial Oncology Society, Royal College of Speech and Language Therapists and NIHR Office for Clinical Research Infrastructure. A pragmatic view was taken on sample size (as no data or precedents are available) and sought to achieve 100 responses from each professional group (physicians and surgeons; dietitians; nurses; speech and language therapists; other). Data were collected anonymously between June 2016 and May 2017.

### Ethical statement

The Research Governance and Quality Assurance Manager from University Hospitals Southampton NHS Foundation Trust’s R&D team confirmed that ethics approval was not considered necessary as the survey was anonymous and did not include any personal information about the participants. Participants consented to take part in the survey by ticking a relevant box on the first page of the survey.

### Statistical analysis

Responses were downloaded from iSurvey to Excel and subsequently imported to SPSS (v23.0, Chicago, IL, USA) for analysis. Descriptive statistical analysis (numbers and percentages) was generated to determine the proportion of respondents who discussed nutrition, confidence in providing nutritional advice, an awareness guidelines, had received training, perceived need for training, areas for training that would support practice as well as the preferred platform of training. Descriptive analyses were carried out using the crosstabs function. As the ‘other’ represented a number of different HCP groups, it was not included in the analysis. Chi square tests were used to assess whether there were associations between the professional groups. Significance was established at *p =* 0.05

## Results

### Responses

There were 610 responses of which there were 191 (31%) nurses (oncology and general), 152 (25%) dietitians (oncology and general) and 187 (31%) doctors (primary care and surgeons/hospital specialists), and of these 79 (13%) were GPs, 54 (9%) speech and language therapists (SLTs) and 26 from ‘other’ groups that included physiotherapists, radiographers, pharmacists and psychologists (Table 2). As the latter group was excluded from analyses, this left a sample of 584 responses. Among those who responded, the HCPs worked with a range of different cancer types. The majority of oncology dietitians specialised in head and neck (42%) and upper GI (43%) cancers, whilst most of the SLTs are specialised in head and neck cancer (75%). Some HCPs specialised in more than one cancer site. The response rate is not known as the survey was sent out to a range of organisations with a wide range of members in addition to existing contacts.

**Table 2:** Numbers of respondents from the professional groups

Professional group	*N* (%)
Nurse (oncology)	148 (24)
Nurse (general)	43 (7)
Dietitian (oncology)	98 (16)
Dietitian (general)	54 (9)
Doctor (secondary and tertiary settings)	108 (18)
GP	79 (13)
Speech and language therapy	54 (9)
Other	26 (4)
Total	610

### Discussion of nutrition

**Fig. 1:**
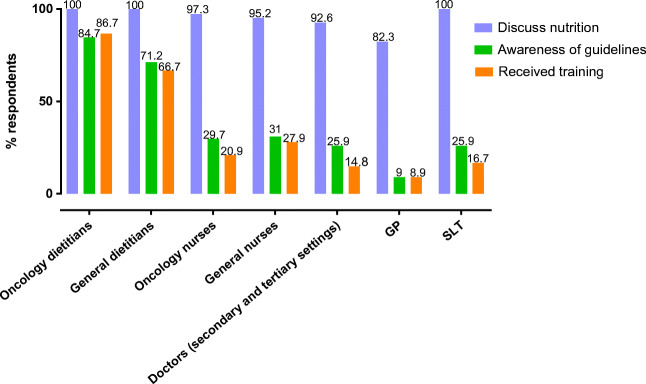
Proportion of respondents by professional group who discussed nutrition, had an awareness of guidelines and received relevant nutrition training

Overall, 94% (*n* = 553) of HCPs discuss nutrition always or sometimes with their patients. All general and oncology dietitians and SLTs discuss nutrition; almost all oncology nurses (97.3%) and general nurses (95.3%) and doctors (92.6%) discuss nutrition but proportionately fewer GPs (82.3%) than other HCP groups (*χ*^2^ = 42.02, df = 6, *p =* 0.001) Fig. 1).

Overall, 77% (*n* = 446) of HCPs provide cancer patients with information on nutrition. As expected, all oncology dietitians provide information, and 94% of general dietitians provide information. Proportionately fewer GPs (*n* = 38, 48.1%) provide information compared with other HCP groups (*χ*^2^ = 117.0, df = 6, *p =* 0.001).

Overall, 63.2% of HCPs provide verbal advice, 43.5% provide local leaflets, 40.5% provide national leaflets, and 16.8% provide information about a website. Proportionately, more oncology dietitians (98.9%), general dietitians (79.0%), oncology nurses (68.9%) and general nurses (60.5%) provide verbal advice in comparison with doctors (48.1%), SLTs (44.4%) and GPs (38%) (*χ*^2^ = 89.78, df = 6, *p =* 0.001). Significantly similar patterns were found for providing local (*χ*^2^ = 171.53, df = 6, *p* = 0.001) and national leaflets (*χ*^2^ = 168.77, df = 6, *p =* 0.001), with fewer GPs (10.1% and 7.6%, respectively) providing these in comparison with other HCPs.

### Awareness of guidelines

**Fig. 2:**
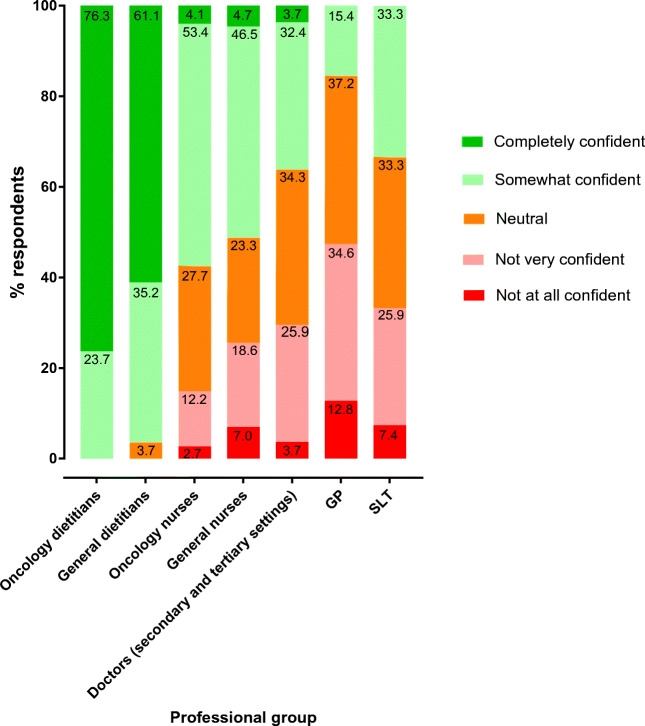
Levels of confidence in providing nutritional advice for each professional group (% respondents)

As a group, 39% (*n* = 226) of the HCPs reported being aware of nutritional guidelines for cancer patients (Fig. 2). Guidelines which were used included Macmillan Cancer Charity (26%), National Institute for Health and Care Excellence (NICE) (25%), European Society for Parenteral and Enteral Nutrition (ESPEN) guidelines (18%), British Dietetic Association (BDA) (10%) and World Cancer Research Fund (WCRF) (8%), as well as guidelines for specific cancer types, e.g. Prostate Cancer UK, ClinicaI Oncology Society of Australia (COSA) Head and Neck guidelines (18%) and other guidelines including local Trust  policy guidelines. Proportionately more oncology dietitians (43.4%) used NICE (*χ*^2^ = 47.06, df = 6, *p =* 0.001) and ESPEN guidelines (39.8%; *χ*^2^ = 36.62 df = 6, *p =* 0.001), and both oncology and general dietitians used BDA guidelines (18.1% and 19.0% respectively, *χ*^2^ = 18.92, df = 6, *p =* 0.001) in comparison with other HCP groups. In contrast, proportionately more oncology nurses used Macmillan information (*χ*^2^ = 33.34, df = 6, *p =* 0.001) in comparison with other groups.

Surprisingly not all of oncology dietitians (85%) and general dietitians (71%) were aware of guidelines. However only 25% of doctors, 30% of nurses, 25% of SLTs and 9% of GPs had an awareness of guidelines (*χ*^2^ = 156.35, df = 6, *p =* 0.001) (Fig. 1). Significantly more HCPs who were aware of the guidelines were found to be completely confident in providing nutritional advice (*n* = 100, 44.4%; *χ*^2^ = 149.50, df = 4, *p =* 0.001) and to always discuss nutrition with their patients (*n* = 177, 79.0%; *χ*^2^ = 79.28, df = 3, *p =* 0.001).

### Nutrition training

Among those that responded, 87% of oncology dietitians and 67% of general dietitians reported having received training for nutritional care for cancer patients. In contrast, 21% of oncology nurses, 28% of general nurses and only 15% of doctors and 9% of GPs had received nutrition training (Fig. 2). More than two-thirds of HCPs who had received training were aware of nutrition guidelines (*n* = 132, 67.3%; *χ*^2^ = 101.28, df = 2, *p =* 0.001).

As expected, oncology and general dietitians all have a degree or PG diploma in nutrition. About two-thirds of oncology and general dietitians report keeping clinically updated through training such as ESPEN, BAPEN, Parenteral and Enteral Nutrition Group *(*PENG), Malnutrition Universal Screening Tool ‘MUST’ training, BDA training and other Continued Professional Development (CPD) courses, through local trust training, reading journals and attending conferences. In contrast, a quarter of oncology nurses reported either ‘MUST’ training, in-house training and training via a CPD course. Three doctors pursed a formal qualification in nutrition, and two had formal ESPEN or ‘MUST’ training. Seven GPs reported ‘MUST’ training, and four taught themselves to use ‘MUST’ screening.

### Confidence in providing nutritional advice and perceived need for training

Overall, 20.4% and 35.4% of all HCPs reported feeling completely or somewhat confident in providing nutritional advice (Fig. 2). Both oncology and general dietitians were most confident (76.3% and 61.1%, respectively) or somewhat confident (23.7% and 35.2%, respectively). Only 3.7% and 32.4% of doctors were completely or somewhat confident in providing nutritional advice. Among oncology nurses, 53.4% weresomewhat confident, but only 4.1% were completely confident. Only 15.4% of GPs were somewhat confident, and none was completely confident. Significantly more HCPs who were aware of guidelines were found to be completely confident in providing nutritional advice (*n* = 100, 44.4%; *χ*^2^ = 149.50, df = 4, *p =* 0.001).

The majority (64.6%, *n* = 376) of HCPs said they have a need for training on nutritional care for cancer patients. Proportionately, more respondents who said they were not very confident, or somewhat confident in providing nutritional advice, wanted training (*n* = 18, 75.0% and *n* = 144, 69.9%, respectively; *χ*^2^ = 59.32, df = 4, *p =* 0.001). Similarly, proportionately more HCPs who had no awareness of guidelines awareness wanted training (*n* = 243, 68.6%; *χ*^2^ = 6.53, df = 1, *p =* 0.05). The greatest need was reported by oncology and general nurses (81%). There were 56% of doctors and 81% of GPs with reported needs for further training as well as 46% of oncology dietitians and 52% of general dietitians. Overall for the professional groups, the identified areas for further training were in order of rank: dietary advice for specific cancer site/stage (70%), assessment of nutrition status (63%), evidence for alternative dietary approaches (57%) and dietary supplements (47%). The preferred approach to provide training was conference/study days (*n* = 399; 68%), face-to-face training (*n* = 340; 58%) and e-learning (*n* = 302; 52%) rather than webinars (*n* = 67; 11%).

Of the 79 GP responses, there were 73 (95%) who considered that GPs have a role to play in supporting the nutritional needs of people living with and beyond cancer. The reasons for their responses were because GPs are the first point of call for patients; they know the patient well, are easily accessible and trusted by the patients, provide a holistic approach to care that includes nutrition and have the responsibility of giving correct information when asked by the patient. Four GPs felt that they did not have a role in supporting nutritional care. They attributed this to existing time constraints on GPs and that they could not be expected to be an expert on everything.

## Discussion

The present study is the largest UK survey that has specifically investigated the provision of nutrition for cancer patients across a wider range of HCP groups. The findings have shown that the majority of HCPs appears to discuss nutrition with their cancer patients, aligning with previous surveys of nutrition support provided by oncologists [13] and for other HCPs where diet was part of lifestyle guidance [16]. Awareness of guidelines varied ranging from using national guidelines and those for specific cancer types of just using utilising trusted sources of guidance.

However, the discussion of nutrition was related to the awareness of guidelines for cancer patients. The awareness of guidelines varied between the different professional groups with most, but not all dietitians having the greatest awareness of guidelines and GPs reporting the least. Those HCPs with a greater awareness of guidelines had received training and were found to be completely confident in providing nutritional advice. Not surprisingly, dietitians were most confident in providing nutritional advice to cancer patients than the other HCP groups, but there were still a fifth of dietitians who were not completely confident. A lack of sufficient time within routine NHS consultations to provide nutritional advice was reported by GPs. Other barriers to providing nutritional advice by HCPs could be attributed to a perceived lack of evidence for the benefit of nutrition interventions, inadequate knowledge and concerns whether the patient is ready and open to receive such advice as well as lack of time [12, 15–18].

Given these reported barriers by HCPs to providing advice, the present study has shown the perceived need for training in the provision of nutritional care for cancer patients across all the professional groups including about half of dietitians but for the majority of nurses, doctors and particularly GPs. The most common aspects that HCPs identified were dietary advice for specific cancer and cancer stage, assessment of nutritional status, alternative dietary approaches and dietary supplements. Thus, education and training in these areas are needed to ensure that cancer patients receive appropriate and consistent advice on nutrition. Consideration should also be given to the way in which education and training are delivered. It should reflect the challenges that HCPs might experience including cost and protected time for continued professional development [8, 19, 20]. In the present study, the preferred platform of training was equally distributed across face-to-face training, conference/study days and E-learning/Webinars. This indicates that both the content and a blended approach to learning should be considered to inform the future design of education and training.

A potential limitation of this study is that the respondents were members of professional organisations and who already may have had an interest in nutrition and more likely to respond. As such this could limit the generalisability of the findings. It was also not possible to determine the exact response rate so may not be representative of all HCPs. However, the proportion of respondents from each professional group was similar for nurses, dietitians and doctors who worked across a range of different cancer specialisms in the UK. More research is needed to explore the potential contribution and role of other HCPs groups that were under-represented in the present study. It would appear that the various professional groups responded differently to the question for awareness of guidelines that included nutritional guidelines as well as sources representing nutritional guidance and information. Further research could explore this aspect with more clarity.

## Conclusions

These findings demonstrate that whilst HCPs provide information on nutrition, awareness of guidelines and confidence in providing nutritional advice are variable. It is important that cancer patients receive consistent evidence-based advice on nutrition from HCPs that is tailored to suit the type and stage of cancer treatment. All HCPs should have access to appropriate nutrition education and training, to improve their knowledge and confidence in providing advice and ensure consistency in practice for quality improvements in patient care. However, it is recognised that there remains a need for more high-quality nutrition research to inform the evidence base to enable HCPs provide appropriate nutritional advice to support cancer care.
